# Rapid detection of *Enterococcus faecalis* using RPA-CRISPR/Cas12a-assisted technology

**DOI:** 10.1128/spectrum.00168-26

**Published:** 2026-05-18

**Authors:** Jiajia Zheng, Zicheng Wen, Yuhua Li, Weihao Zou, Ping Ge, Hongjuan Peng

**Affiliations:** 1 Department of Pathogen Biology, Guangdong Provincial Key Laboratory of Tropical Diseases Research, School of Public Health, Key Laboratory of Infectious Diseases Research in South China (Southern Medical University), Ministry of Education, Southern Medical University 70570, Guangzhou city, Guangdong Province, People's Republic of China; 2 Department of Clinical Laboratory, Armed Police Corps Hospital of Guangdong Province, Guangzhou City, Guangdong Province, People's Republic of China; Institut National de Santé Publique du Québec, Sainte-Anne-de-Bellevue, Québec, Canada

**Keywords:** *Enterococcus faecalis*, recombinase polymerase amplification, CRISPR/Cas12a, nucleic acid detection, rapid detection

## Abstract

*Enterococcus faecalis* (*E. faecalis*) is an opportunistic pathogen capable of causing various life-threatening infections, including urinary tract infections, bloodstream infections, infective endocarditis, and meningitis. As a major etiological agent of healthcare-associated infections (HAIs), its global prevalence continues to rise, a trend closely linked to the increasing problem of multidrug resistance driven by overuse of antibiotics. Therefore, rapid and accurate detection is essential for timely treatment and improved prognosis. In this study, the *pheS* gene of *E. faecalis* was rapidly amplified using recombinase polymerase amplification (RPA), and detection was achieved via a CRISPR/Cas12a system. The Cas12a-crRNA complex specifically recognized the amplification product and triggered nonspecific cleavage of a single-stranded DNA (ssDNA) reporter, generating a fluorescent signal that could be quantified in a real-time PCR system or visualized directly under ultraviolet (UV) light. After optimization of key parameters—including RPA primers, reaction conditions, crRNA sequence, and the crRNA/Cas12a combination—the assay achieved a limit of detection (LOD) of 10^−2^ ng/μL within a short turnaround time, and showed no cross-reactivity with other common pathogen detection results from clinical isolates and spiked samples were fully consistent with those obtained through PCR/qPCR, confirming high reliability. In summary, the RPA-CRISPR/Cas12a detection method established in this study is sensitive, specific, and reliable. Its simplicity, minimal equipment requirements, and cost-effectiveness make it a promising tool for rapid clinical detection of *E. faecalis*.

**IMPORTANCE:**

*Enterococcus faecalis* is a major opportunistic pathogen responsible for severe healthcare-associated infections, with rising prevalence linked to antibiotic resistance. Rapid and accurate detection is critical for timely treatment and infection control. Conventional methods are often time-consuming or require complex laboratory infrastructure, limiting their use at the point of care. This study developed a rapid detection assay by integrating recombinase polymerase amplification with the CRISPR/Cas12a system, targeting the *pheS* gene of *E. faecalis*. The method is sensitive and specific, providing visual results under UV light within a short turnaround time. It offers a simple, cost-effective, and requires minimal equipment, suitable for clinical and resource-limited settings, potentially improving diagnostic efficiency and supporting antimicrobial stewardship.

## INTRODUCTION

*Enterococcus faecalis* is a common opportunistic pathogen and a predominant gram-positive bacterium within *Enterococcus* spp. It produces various virulence factors, including lipoteichoic acid (1), gelatinase, serine proteases (2, 3), peptidoglycan, and cytolysin (4, 5), which facilitate its adhesion, colonization, and pathogenesis in diverse host environments. *Enterococcus* spp. have emerged as one of the leading causes of healthcare-associated infections (HAIs), with *E. faecalis* alone accounting for approximately 60% of all enterococcal infections (6). Recent studies have demonstrated that it can be responsible for up to 80–90% of such infections, ranking only behind *Escherichia coli* and *Staphylococcus* spp. as a major global cause of nosocomial infections (7). *Enterococcus* spp. rank as the third leading cause of infective endocarditis in high-income countries, with *E. faecalis* alone responsible for over 90% of these cases and associated with higher rates of heart failure and splenic abscesses, particularly among elderly patients with significant comorbidities (8). Furthermore, *E. faecalis* has emerged as the second most prevalent causative agent of meningitis, trailing only *Staphylococcus aureus*, with a continually rising incidence (9, 10). The clinical impact of *E. faecalis* is exacerbated by the rising prevalence of multidrug-resistant strains, a trend driven by prolonged hospital stays, immunocompromised patients, and antibiotic misuse (11). This has led to increased treatment costs, morbidity, and mortality rates (12). Therefore, it is urgently necessary to develop diagnostic methods that are not only accurate but also rapid enough to guide timely therapy and infection control.

Conventional pathogen detection methods encompass bacterial isolation and culture, serological assays, and molecular biology techniques (13). Bacterial culture remains the gold standard for pathogen diagnosis; however, it is time-consuming (14), and its effectiveness depends on the type of culture media used (15), which presents certain limitations. Enzyme-linked immunosorbent assay (ELISA), based on specific antigen-antibody binding, enables effective detection through absorbance measurement, yet its relatively high cost and pronounced cross-reactivity restrict its broader application (16). Molecular techniques such as polymerase chain reaction (PCR) and quantitative real-time PCR (qPCR) exhibit high sensitivity and specificity. Nevertheless, these methods require expensive instrumentation, sophisticated experimental procedures, and stringent laboratory conditions (17), limiting their use at the point of care.

Novel isothermal amplification techniques, such as recombinase polymerase amplification (RPA) and loop-mediated isothermal amplification (LAMP) (18), enable target gene amplification at constant temperatures. Compared to conventional PCR, these methods offer notable advantages, including compatibility with simple heating devices, straightforward operation (19), mild temperature requirements, and rapid reaction kinetics (20). These features have facilitated the broader adoption of molecular diagnostics and demonstrate considerable promise for point-of-care testing (POCT). RPA, as one of the isothermal nucleic acid amplification approaches, employs recombinase-primer complexes to scan double-stranded DNA for homologous sequences, initiate strand exchange, and subsequently trigger DNA synthesis, resulting in exponential amplification of the target sequence. However, a major drawback of RPA is its propensity for nonspecific amplification, which leads to false-positive results (21), thereby constraining its achievable sensitivity and specificity.

The CRISPR-Cas system, a rapidly advancing gene-editing technology, has become a powerful platform for nucleic acid detection due to its speed, high sensitivity, and specificity (22). Based on the key characteristics of Cas effectors, the system is broadly divided into three classes, including Cas9, Cas12, and Cas13. Among these, Cas12a is a crRNA-guided endonuclease that, upon recognition of a T-rich protospacer adjacent motif (PAM), guides the target double-stranded DNA (dsDNA) to the catalytic domain for cleavage (23). Initially, under the guidance of crRNA targeting the specific sequence, Cas12a first exhibits cis-cleavage activity by specifically cleaving the target dsDNA. This activation then triggers trans-cleavage activity, leading to the nonspecific cleavage of surrounding single-stranded DNA (ssDNA) reporters (24, 25). This cleavage severs the linkage between the quencher and fluorophore, resulting in the generation of a fluorescent signal. However, the relatively low trans-cleavage efficiency of Cas12a and the difficulty in detecting low target concentrations make a pre-amplification step necessary (26). To address this, Chen et al. developed the DNA Endonuclease Targeted CRISPR *Trans* Reporter (DETECTR) system by integrating RPA with CRISPR/Cas12a (27), which significantly improved the sensitivity and specificity of nucleic acid detection and advanced molecular testing. To date, the RPA-CRISPR/Cas12a combination technology has been successfully applied to detect diverse pathogens, such as *Staphylococcus aureus* (28), *Bacillus anthracis* (29), *Salmonella* spp. (30), and monkeypox virus (31).

Given the significant clinical impact of *Enterococcus faecalis*, RPA-CRISPR/Cas12a technology has also been successfully applied in its detection. Tu et al. targeted the *sodA* gene of *E. faecalis* and the *endA* gene of *Escherichia coli* to develop an integrated platform (ME-CRISPR) combining magnetic enrichment, photothermal lysis, and duplex RPA-CRISPR. This system enables the simultaneous detection of both pathogens in urine samples, with a limit of detection of 10 CFU/mL and a total assay time of 40 min (32), thereby providing an important foundation for the rapid detection of *E. faecalis* and related pathogens. Meanwhile, *E. faecalis* is also widely used as a negative control for the detection of other bacteria using the RPA-CRISPR/Cas12a system. Chen et al. integrated this system into a centrifugal-driven microfluidic chip for rapid detection of *Salmonella* spp., with *E. faecalis* serving as a negative control to validate specificity (33). Zhang et al. developed an amplification-free electrochemiluminescent biosensor based on CRISPR/Cas12a for ultrasensitive detection of *Fusobacterium nucleatum*, in which *E. faecalis* was used exclusively as a negative control to assess specificity (34).

Regarding the method developed by Tu et al., it utilizes a one-pot duplex RPA-CRISPR/Cas12a-Cas13a system, in which *E. faecalis* detection relies on the Cas13a system, while *E. coli* detection employs Cas12a. Furthermore, this approach requires specialized nanomaterials and near-infrared laser instrumentation, which may limit its broader application in resource-limited settings.

In this research, we aim to develop a rapid nucleic acid detection method yielding visually interpretable results by combining RPA with CRISPR/Cas12a. This approach is expected to provide a novel, practical tool for early identification of *E. faecalis* infections in clinical settings, supporting timely intervention and informed antimicrobial stewardship amid growing challenges of antibiotic resistance.

## MATERIALS AND METHODS

### Materials and reagents

All commercial kits and reagents were used according to manufacturers' instructions. The CRISPR/Cas12a DNA Detection Kit was purchased from Yizhi Biotechnology Co., Ltd. (Guangdong, China). The Universal DNA Extraction Mini Kit was obtained from Fangzhou Biosafety Technology Co., Ltd. (Guangdong, China). The SYBR Green Master Mix for qPCR was sourced from Yeasen biotech Co., Ltd. (Shanghai, China). TEV protease was acquired from New England Biolabs (Guangdong, China), and nuclease-free water was purchased from TransGen Biotech Co., Ltd. (Beijing, China). Oligonucleotides, including RPA primers and crRNA, were synthesized by Sangon Biotech Co., Ltd. (Shanghai, China) and Tsingke Biotech Co., Ltd. (Beijing, China), respectively. The plasmid 6His-MBP-TEV-huLbCpf1 was also synthesized by Sangon Biotech Co., Ltd. The LbCas12a protein was expressed and purified in our laboratory.

### Bacterial strains and clinical specimens

A total of 12 clinical patient-derived *E. faecalis* isolates and 6 other common pathogenic strains (*Acinetobacter baumannii*, *Proteus* spp., *Staphylococcus sciuri*, *Klebsiella pneumoniae*, *Enterococcus faecium*, and *Escherichia coli*) were all obtained from a hospital in Guangdong Province, China. The isolates were initially cultured on blood agar plates, and species identification was performed using the VITEK 2 Compact automated identification and susceptibility testing system (bioMérieux, France) following the manufacturer’s instructions. Nineteen urine samples from healthy adults were also collected. All isolates were identified and stored in cryopreservation tubes at −80°C.

### Workflow of the rapid *E. faecalis* detection method

**Fig 1:**
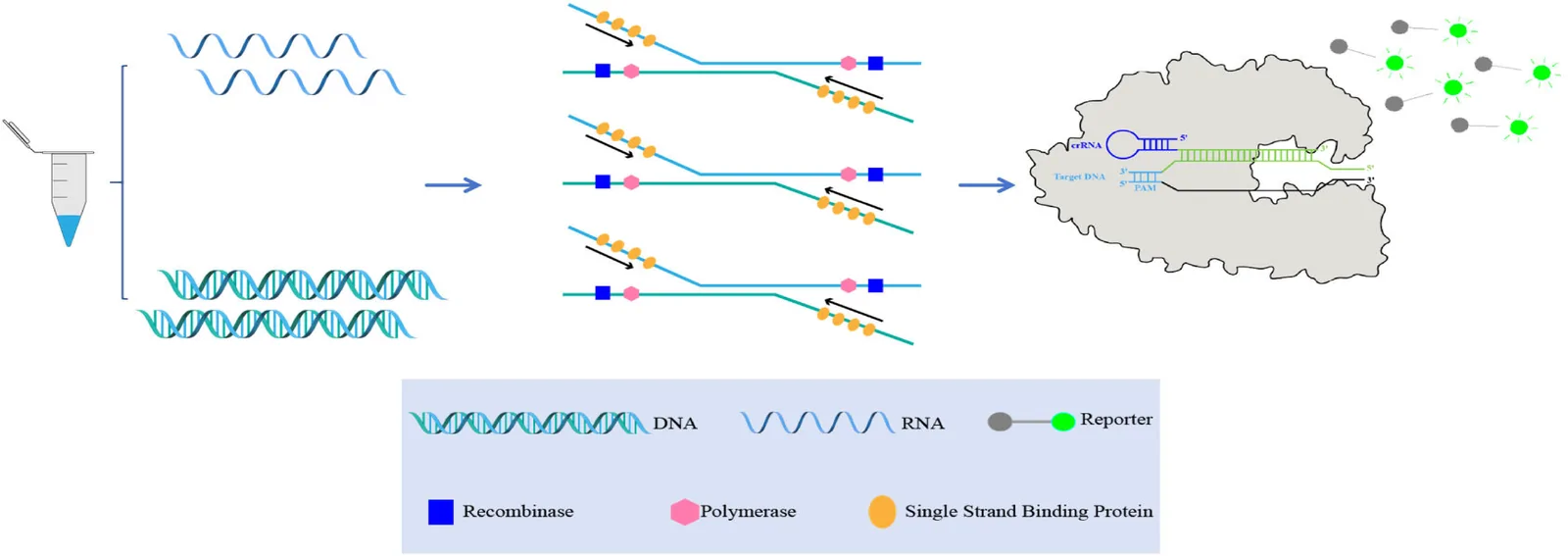
Workflow of the rapid *E. faecalis* detection method. Sample DNA is extracted, then amplified by RPA and identified by a CRISPR-Cas12a system that transduces the target sequence into a visible fluorescent signal.

The detection workflow of the RPA-CRISPR/Cas12a-based assay for *E. faecalis* is illustrated in Fig. 1. First, bacterial genomic DNA is extracted from the sample. The target sequence is then amplified isothermally via RPA. The resulting amplicon is combined with the CRISPR/Cas12a detection system. Upon specific target recognition, the activated Cas12a-crRNA complex cleaves single-stranded DNA reporters, releasing fluorophores from quenchers and generating a fluorescent signal. This fluorescence can be measured in a real-time PCR instrument or observed directly under UV light. A positive result is confirmed by the presence of fluorescence, while its absence indicates a negative result.

### Primer and crRNA design

The *pheS* gene sequence (GenBank: AJ843387.1) of *E. faecalis* was obtained from the NCBI database. RPA primers were designed using the EZ Assay online design tool (https://ezassay.com/primer), and their specificity was verified by NCBI BLAST.

crRNAs targeting the amplified sequences were designed using the EZ Assay crRNA design platform (https://www.ezassay.com/rna?source=bing). Each crRNA was composed of a target-specific spacer sequence and the direct repeat sequence (5′-UAAUUUCUACUAAGUGUAGAU-3′). The detailed information on primers and crRNAs involved in this study was summarized in Table 1.

**TABLE 1:** Sequence of primers and crRNAs

Name	Sequence (5′−3′)
*E. faecalis*-F1	ACATAGTCACCAATTCCATCAGATTGAAG
*E. faecalis*-R1	GCAGACGTTACAACCTGCGCCGCCACATT
*E. faecalis*-F2	CATAGTCACCAATTCCATCAGATTGAAGGCC
*E. faecalis*-R2	GATGGAATTGGTGACTATGTGTCGCATCATC
*E. faecalis*-F3	ACACATAGTCACCAATTCCATCAGATTGAAGG
*E. faecalis-*R3	GCAGACGTTACAACCTGCGCCGCCACATTTAA
*E. faecalis*-F4	TACAGATGATGCGACACATAGTCACCAATTCC
*E*. *faecalis*-R4	CAACCTGTATGTTTGCAGACGTTACAACCTGC
*E. faecalis*-F5	CTCCTGGGAAAGTATTCCGTCGTGATACAGA
*E. faecalis*-R5	AAAGGGAAATAGCTAGGACGTAAACGAATTT
*E. faecalis*-F6	CCGTCGTGATACAGATGATGCGACACATAGTCA
*E. faecalis*-R6	AACAGCTAACATCTACTTCGACTGAAGGTTCTG
*E. faecalis*-F7	GATGATGCGACACATAGTCACCAATTCCATC
*E. faecalis*-R7	AACAGCTAACATCTACTTCGACTGAAGGTTC
*E. faecalis*-crRNA-1	UAAUUUCUACUAAGUGUAGAUGUGAAGAUCGUAAAAUUCGUUUA
*E. faecalis*-crRNA-2	UAAUUUCUACUAAGUGUAGAUAAAGGGACGUUAGAAGUCAUGAU

### Expression and purification of recombinant LbCas12a protein

Recombinant LbCas12a protein was expressed and purified as follows. *Escherichia coli* BL21(DE3) harboring the 6His-MBP-TEV-huLbCpf1 plasmid was grown in LB medium at 37°C to an OD_600_ of 0.6–0.8. Then, protein expression was induced with IPTG and the LbCas12a protein was subsequently purified via affinity chromatography using a stepwise imidazole gradient for elution. The purified fusion protein was then digested with TEV protease to remove the MBP tag, followed by concentration. The expression level and purification efficiency were assessed by SDS-PAGE and Coomassie Brilliant Blue staining.

### Bacterial culture and genomic DNA extraction

*E. faecalis* from cryopreservation tubes was streaked onto blood agar plates and incubated at 37°C for 24 h. A single colony was then inoculated into 5 mL of LB broth and cultured at 37°C with shaking (200 rpm) for 12 h. Genomic DNA from bacterial cultures and urine samples was extracted using the Universal DNA Extraction Mini Kit according to the manufacturer’s instructions.

### Recombinase polymerase amplification

RPA was performed in a 10 μL reaction containing: 5 μL of 2× Reaction Buffer, 1 μL of 10× P-mix, 1 μL of 10× E-mix, 0.25 μL of each Forward and Reverse Primer (20 μM), 0.5 μL of DNA template, and 1 μL of Nuclease-free H_2_O. Finally, 1 μL of 10× Starter was added. The tube was gently flicked to mix, briefly centrifuged to remove bubbles, and incubated at 39°C for 40 min in a PCR instrument with the heated lid turned off.

### CRISPR-Cas12a cleavage assay

The Cas12a detection was performed in a 10 μL reaction containing: 1 μL of 10× Cleavage Buffer, 0.3 μL of 4 μM Reporter (ssDNA-FQ), 0.5 μL of Cas12a Protein, 0.5 μL of 1 μM crRNA, 0.5 μL of RPA amplicon, and 7.2 μL of Nuclease-free H_2_O. After gentle mixing by flicking the tube, the reaction tube was placed in a real-time PCR instrument. Fluorescence intensity was recorded every two minutes during a one-hour incubation at 37°C. Upon completion, the reaction products were visualized and imaged under UV light using a smartphone to capture the fluorescence.

### Optimized reaction conditions for the RPA-CRISPR/Cas12a assay

To establish the optimal reaction conditions for the RPA-CRISPR/Cas12a assay, a systematic optimization was performed. First, the RPA amplification step was optimized by testing different reaction temperatures (39°C, 40°C, and 41°C) and amplification times (20, 40, and 60 min). The amplification products were analyzed by 1.5% agarose gel electrophoresis to determine the most efficient conditions. Furthermore, to maximize the detection efficiency of the Cas12a cleavage step, the concentrations of both the Cas12a protein and crRNA were optimized. Combinations with four concentration levels (10, 30, 50, and 100 nM) for each component were employed. The optimal combination was determined by comparing the fluorescence intensities obtained from each reaction.

### Specificity and sensitivity assessment

To evaluate the specificity of the established assay, genomic DNA from six clinically common pathogens was extracted and then subjected to the optimized RPA-CRISPR/Cas12a detection method. The fluorescence results were recorded and visualized under UV light.

The sensitivity of the method was determined by testing a 10-fold serial dilutions of *E. faecalis* genomic DNA, with concentrations ranging from 1.21 × 10^1^ to 1.21 × 10^−4^ ng/µL. Each dilution was tested in triplicate, and the fluorescence results were recorded and visualized under UV light.

### Clinical strains detection

To evaluate the clinical utility of the established method for detecting *E. faecalis* from diverse clinical sources, a total of 12 *E. faecalis* strains isolated from different patients were analyzed in parallel using the optimized RPA-CRISPR/Cas12a assay and conventional PCR, and the results were compared. The 25 μL PCR reaction mixture contained 12.5 μL of 2× Phanta Flash Master Mix, 1 μL of Forward Primer (10 μM), 1 μL of Reverse Primer (10 μM), 0.5 μL of DNA template, and 10 μL of Nuclease-free H_2_O. The PCR cycling program consisted of an initial denaturation at 98°C for 30 s, followed by 35 cycles of denaturation at 98°C for 10 s, annealing at 58°C for 5 s, extension at 72°C for 2 s, and a final extension at 72°C for 1 min. Subsequently, the PCR amplification products were analyzed by 1.5% agarose gel electrophoresis.

### Clinical samples detection

Ten spiked urine samples were prepared by spiking 100 μL of a 1 × 10^6^ CFU/mL *E. faecalis* culture into 900 μL of healthy urine, achieving a final concentration of 1 × 10^5^ CFU/mL. Conversely, nine control samples were prepared by adding 100 μL of ddH_2_O to 900 μL of urine. Bacterial DNA from all samples was extracted and analyzed in parallel using the optimized RPA-CRISPR/Cas12a assay and qPCR. The qPCR amplification was carried out in a 20 μL mixture consisting of 10 μL SYBR Green Master Mix, 0.4 μL of each Primer (10 μM), 1 μL of Template DNA, and 8.2 μL Nuclease-free H_2_O. The thermal cycling protocol included an initial denaturation at 95°C for 5 min, followed by 40 cycles of 95°C for 10 s and 58°C for 30 s.

### Statistical analysis

Data were analyzed using SPSS 20 and visualized using GraphPad Prism 9.5. Statistical significance was determined by Student’s *t*-test or one-way analysis of variance (ANOVA), with *P* < 0.05 considered statistically significant.

## RESULTS

### Establishment and optimization of the RPA assay

**Fig 2:**
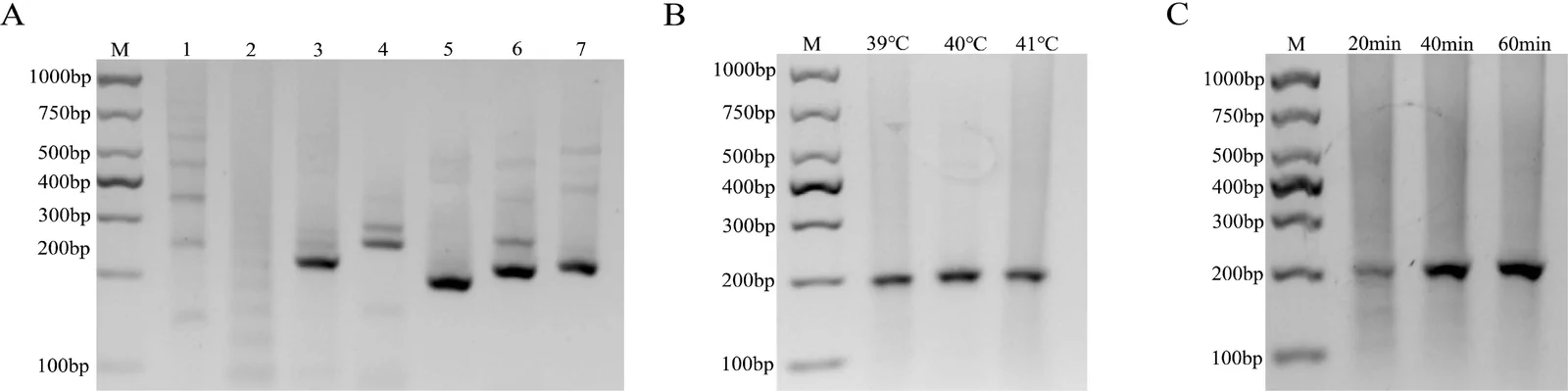
Establishment and optimization of the RPA assay for *E. faecalis* detection. (**A**) Screening of RPA primer pairs targeting the *pheS* gene. Lanes 1–7 show amplification products generated by primer pairs F1/R1 to F7/R7. (**B and C**) Optimization of reaction conditions using the F5/R5 primer set. (**B**) Effect of reaction temperature (39°C, 40°C, and 41°C). (**C**) Effect of incubation time (20, 40, and 60 min). M, DL 1000 DNA marker.

To develop a rapid detection method for *E. faecalis*, an RPA assay targeting the *pheS* gene was established and optimized. Initially, seven pairs of RPA primers were designed and synthesized. Following amplification, products were incubated at 65°C for 10 min and then analyzed by 1.5% agarose gel electrophoresis at 140 V for 40 min. The results revealed that three primer pairs, F3/R3, F4/R4, and F5/R5, successfully amplified the target sequence, generating amplicons of the expected size (Fig. 2A). Among these, the F5/R5 primer pair yielded the brightest band with minimal nonspecific products, demonstrating superior amplification efficiency (Fig. 2A).

We optimized the RPA reaction conditions for the selected F5/R5 primer set by testing a range of temperatures (39°C, 40°C, and 41°C) and reaction times (20, 40, and 60 min). Products were analyzed by 1.5% agarose gel electrophoresis (Fig. 2B and C). Amplification was detectable at 20 min, with band intensity increasing progressively over time, and distinct bands were achieved at both 40 and 60 min. Regarding temperature, the strongest band intensity was achieved at 40°C. As a result, balancing assay rapidity with robust signal detection, we selected 40°C and 40 min as the optimal RPA condition for subsequent experiments.

### Establishment and optimization of the CRISPR-Cas12a detection system

**Fig 3:**
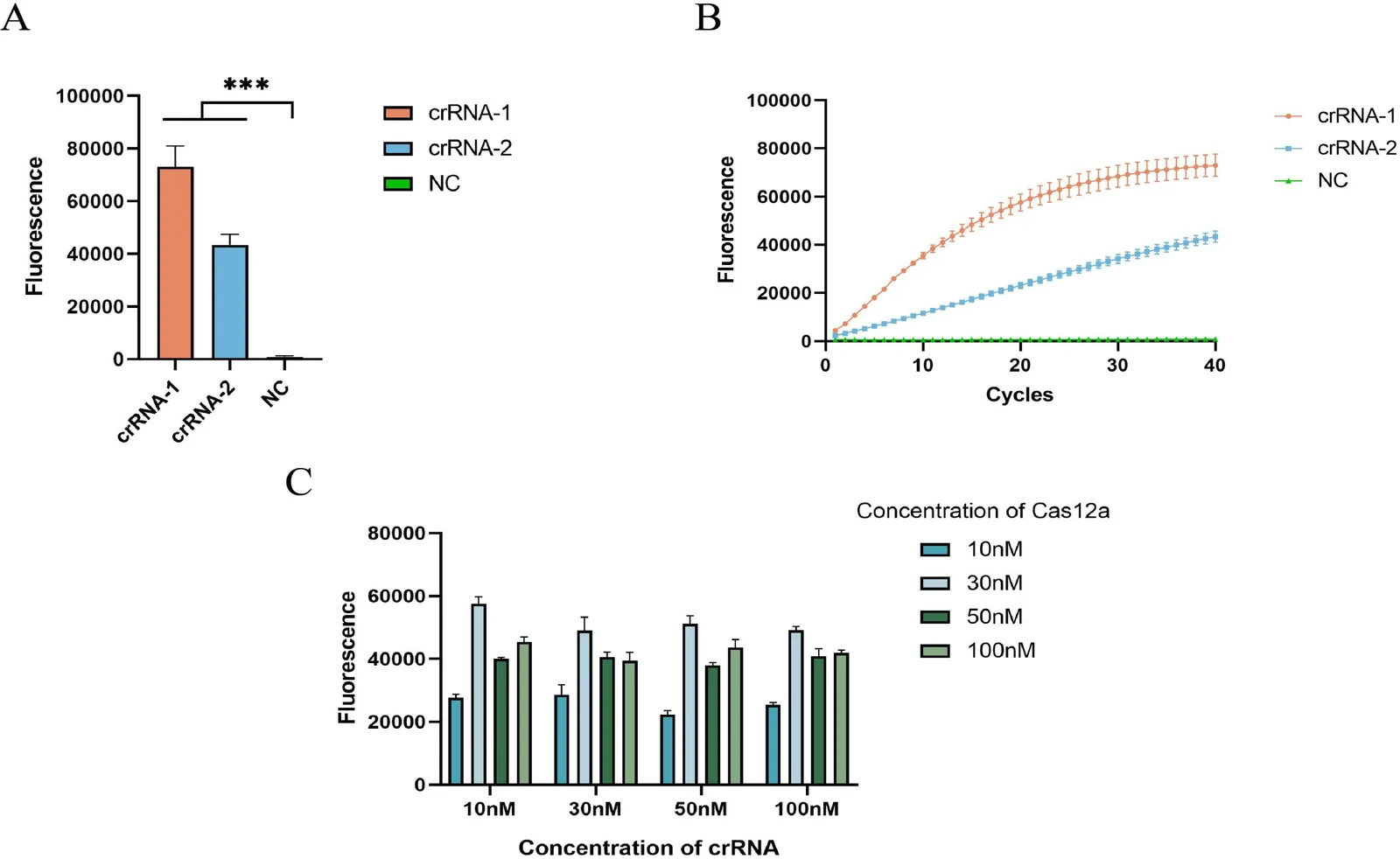
Establishment and optimization of the CRISPR-Cas12a detection system for *E. faecalis*. (**A**) Comparison of endpoint fluorescence intensity and corresponding visual fluorescence under UV light across different groups (***P < 0.001). (**B**) Real-time fluorescence curves during the Cas12a cleavage reaction guided by different crRNAs. (**C**) Optimization of Cas12a and crRNA concentrations was performed using endpoint fluorescence. Data are presented as mean ± SD deviation (*n* = 3). NC, negative target control.

The selection of the crRNA targeting site is a key factor influencing the cleavage efficiency of Cas12a (35). To identify the most effective guide RNA for detection, two distinct crRNAs targeting different regions of the *pheS* gene were designed. Their performance was assessed by monitoring real-time fluorescence upon target recognition. Although both crRNAs enabled specific Cas12a-mediated cleavage, crRNA-1 generated approximately twofold higher fluorescence intensity than crRNA-2, indicating superior reaction efficiency (Fig. 3A and B). Therefore, crRNA-1 was selected for the construction of the CRISPR-Cas12a detection system.

To maximize the detection efficiency of the Cas12a cleavage system, we systematically tested different concentration combinations of the Cas12a protein and crRNA. Each component was evaluated at 10, 30, 50, and 100 nM in all possible combinations. The endpoint fluorescence signal was measured to evaluate the performance of each combination. The combination of 30 nM Cas12a and 10 nM crRNA yielded the strongest fluorescence signal, and was established as the optimal condition for the assay (Fig. 3C).

### Specificity and sensitivity of the RPA-CRISPR/Cas12a assay

**Fig 4:**
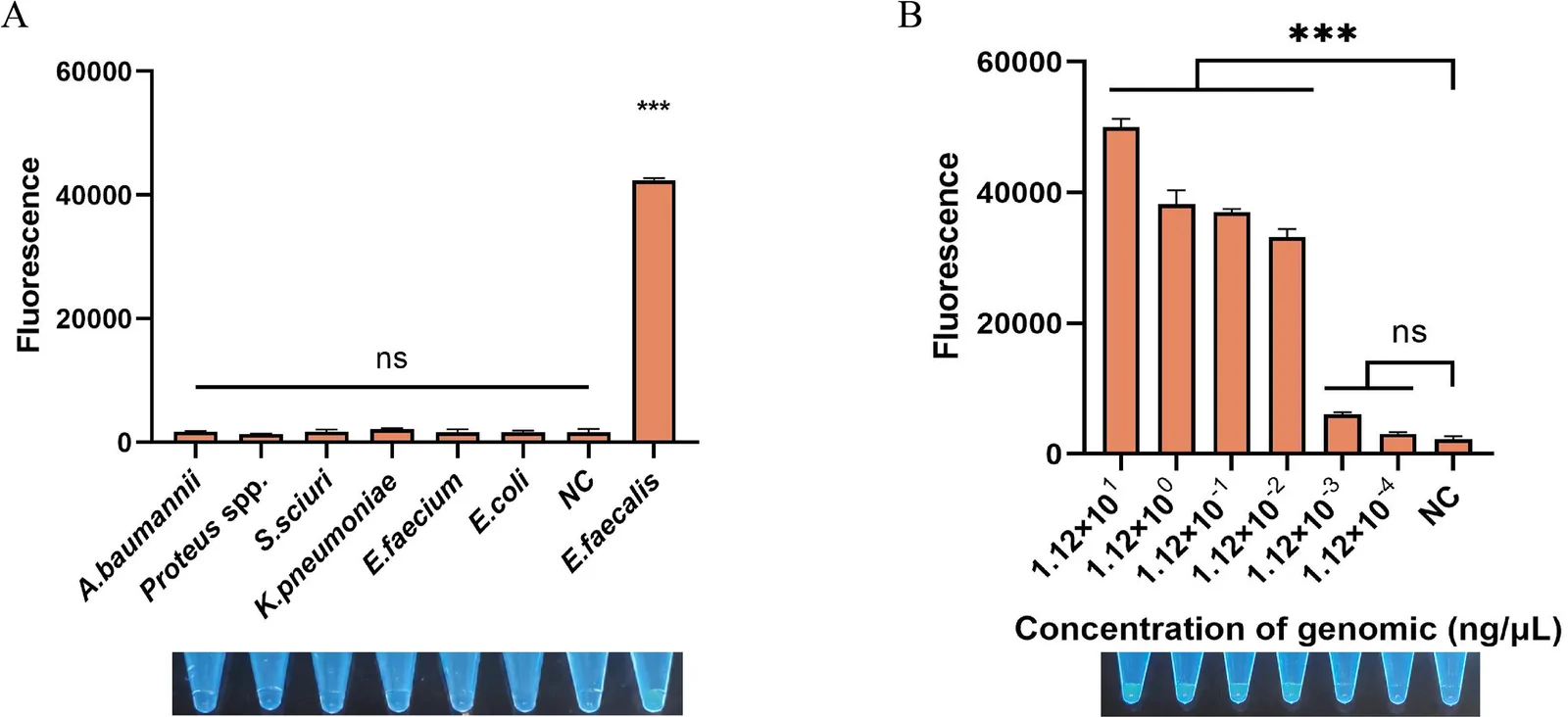
Specificity and sensitivity of the RPA-CRISPR/Cas12a assay for *E. faecalis* detection. (**A**) Specificity was assessed using genomic DNA from *E. faecalis* and six clinically common pathogens. (**B**) Sensitivity was determined using 10-fold serial dilutions of *E. faecalis* genomic DNA (10^1^–10^−4^ ng/μL). Data are presented as mean ± standard deviation (*n* = 3). Statistical significance was determined by one-way ANOVA followed by Dunnett’s test. NC, negative target control; ***P < 0.001, ns: no significant difference.

The specificity of the established RPA-CRISPR/Cas12a assay was evaluated using genomic DNA from six clinically common pathogens, including *Acinetobacter baumannii*, *Proteus* spp., *Staphylococcus sciuri*, *Klebsiella pneumoniae*, *Enterococcus faecium*, and *Escherichia coli*. As shown in Fig. 4A, a strong positive fluorescence signal was observed only for *E. faecalis*. In contrast, all non-target samples and the negative target control (NC) showed only background-level fluorescence, with the difference being statistically significant (*P* < 0.001). These results confirm the high specificity of the assay and the absence of cross-reactivity with the tested bacterial species.

To determine analytical sensitivity, 10-fold serial dilutions of *E. faecalis* genomic DNA (from 10^1^ to 10^−4^ ng/μL) were tested. Fluorescence intensity decreased correspondingly with template concentration (Fig. 4B). Distinct positive signals were consistently generated at DNA concentrations down to 10^−2^ ng/μL (Fig. 4B). A marked decline in signal was observed at concentrations below this threshold (Fig. 4B). The assay consistently detected DNA down to 10⁻² ng/μL, establishing this as the limit of detection (LOD).

### Clinical strains detection

**Fig 5:**
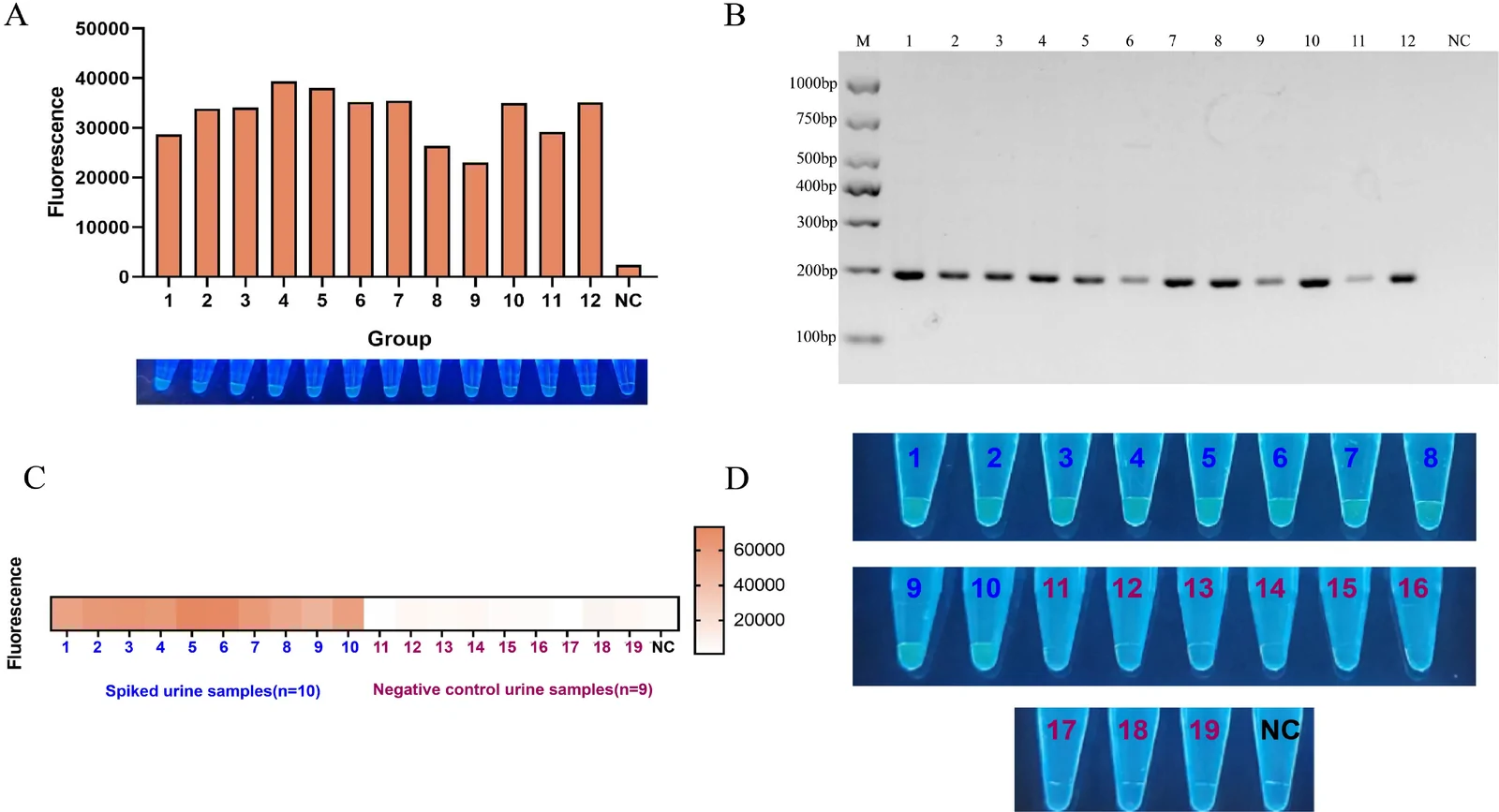
Clinical detection of RPA-CRISPR/Cas12a method. (**A**) Clinical validation of the RPA-CRISPR/Cas12a assay using 12 clinical *E. faecalis* isolates. Detection results showing the endpoint fluorescence intensity and corresponding visual fluorescence under UV light for all 12 strains. (**B**) Agarose gel electrophoresis of PCR amplification products from the same 12 isolates, confirming the presence of the target *pheS* gene amplicon. (**C**) Heatmap of fluorescence intensity values for the 19 tested urine samples. (**D**) Visual fluorescence of the corresponding urine Samples under UV light. Samples 1–10: *E. faecalis*-spiked urine samples; Samples 11–19: negative control urine samples. M, DL 1000 DNA marker; NC, negative target control (Table 2).

To evaluate diagnostic performance of the established assay, 12 *E. faecalis* isolates obtained from diverse clinical specimens were tested using both the RPA-CRISPR/Cas12a assay (Fig. 5A) and conventional PCR (Fig. 5B). All 12 isolates were positive by using both methods, demonstrating complete concordance (100%) between the two methods (Fig. 5A and B). These results verify robust detection capability of the method for clinically derived *E. faecalis* isolates.

### Clinical samples detection

Ten samples were artificially spiked with *E. faecalis* at a concentration of 10^5^ CFU/mL, while the remaining nine samples were spiked with an equal volume of ddH_2_O to serve as negative controls. All samples were tested in parallel using both the established RPA-CRISPR/Cas12a method and qPCR. The results showed complete agreement between two methods, with all ten spiked samples were correctly identified as positive, and all nine negative controls were correctly identified as negative (Fig. 5C and D). These findings demonstrate that the proposed method performs comparably to qPCR in a clinical sample setting, supporting its potential for diagnostic application.

**TABLE 2:** RPA-CRISPA-Cas12a and qPCR for spiked urine samples

		qPCR		
		Positive	Negative	Total
RPA-CRISPR/Cas12a	Positive	10	0	10
	Negative	0	9	9
	Total	10	19	19

## DISCUSSION

In this study, we successfully established and optimized a rapid detection method for *E. faecalis* by integrating RPA with the CRISPR/Cas12a system. This assay targets the *pheS* gene, which is rapidly amplified by RPA. The resulting amplicons are then recognized by a Cas12a-crRNA complex, activating its trans-cleavage activity to cleave fluorescent reporters and generate a detectable signal. The entire process, completed within 2 h, achieves a detection limit of 10^−2^ ng/μL and exhibits no cross-reactivity with other common pathogenic bacteria, confirming high specificity. A significant advantage of this method is its flexibility in result interpretation: fluorescence can be recorded using a real-time PCR instrument or directly visualized as green fluorescence under UV light, making it highly suitable for point-of-care testing (POCT). Furthermore, when evaluated with spiked urine samples to simulate clinical contamination, the assay demonstrated perfect concordance with qPCR results, attesting to its reliability.

In recent years, multiple studies have applied RPA-CRISPR technology to the detection of *E. faecalis*. The microfluidic chip developed by Chen et al. (33) for rapid detection of *Salmonella* and the electrochemical biosensor developed by Zhang et al. (34) for ultrasensitive detection of *Fusobacterium nucleatum* both employed *E. faecalis* as a negative control strain to validate assay specificity, providing a reference for the subsequent development of RPA-CRISPR/Cas12a methods specifically targeting *E. faecalis*.

Compared with the ME-CRISPR integrated platform developed by Tu et al. (32), the RPA-CRISPR/Cas12a assay established in this study exhibits distinct positioning and advantages in several aspects. First, regarding target gene selection, Tu et al. targeted the *sodA* gene of *E. faecalis*, whereas this study, for the first time, employed the *pheS* gene as the detection target. This gene shows no cross-reactivity with other clinically common pathogenic bacteria, and the panel of strains used for specificity testing in this study was essentially consistent with that of Tu et al., confirming the favorable specificity of this method. Furthermore, Tu et al. required prior transcription of the amplicon into RNA via T7 transcription, followed by recognition and detection using Cas13a system. In contrast, this study directly employs the Cas12a system to detect DNA amplicons, eliminating the need for transcription, reducing operational steps, and simplifying the reaction procedure. Additionally, in terms of equipment requirements, the method of Tu et al. relies on specialized nanomaterials and near-infrared laser instrumentation, which limits its application in resource-limited settings. In contrast, this study only requires basic laboratory equipment, such as a real-time PCR instrument or UV light, making it more suitable for point-of-care testing or use in resource-limited regions. Therefore, while meeting clinical diagnostic requirements, this method offers significant advantages in simplicity and accessibility, providing a practical alternative for the rapid detection of *E. faecalis* in resource-limited settings.

However, this study has certain limitations. First, the assay primarily serves as a qualitative or semi-quantitative tool and lacks the precise quantitative capability of qPCR. Second, in terms of clinical validation, while the use of artificially spiked samples effectively demonstrates the method’s specificity and anti-interference potential, the relatively small sample size and absence of diverse real clinical samples mean it may not fully represent the clinical complexity. Therefore, further studies should aim to collect a larger number of real clinical samples from diverse sources, such as blood and urine, to comprehensively evaluate the clinical sensitivity, specificity, and predictive value of this diagnostic method. Furthermore, since RPA amplification and CRISPR cleavage are performed as two independent steps, the transfer of RPA products for Cas12a detection requires tube opening, which carries a risk of aerosol contamination and may increase the false-positive rate. It is necessary to optimize this procedure to minimize contamination risks. Ongoing research is to explore strategies such as physical separation of RPA and Cas12a systems (36, 37) and light-activated Cas12a-assisted RPA detection to achieve one-step detection (38).

In future studies, we will focus on expanding the clinical sample size to enhance statistical power, as well as prioritizing the development of an integrated one-pot reaction system, which advances this technology toward more robust and efficient clinical application.

### Conclusion

In summary, we have established a rapid, specific, and equipment-flexible RPA-CRISPR/Cas12a assay for *E. faecalis* detection. This method provides a practical tool for the early identification of this important pathogen and holds promise for improving diagnostic turnaround time in clinical and resource-limited settings. Further development toward a one-pot format and expanded clinical validation will be important steps toward its translational applications.

## Data Availability

All data generated or analyzed during this study are included in this published article.
